# Plasmid ATLAS: plasmid visual analytics and identification in high-throughput sequencing data

**DOI:** 10.1093/nar/gky1073

**Published:** 2018-11-05

**Authors:** Tiago F Jesus, Bruno Ribeiro-Gonçalves, Diogo N Silva, Valeria Bortolaia, Mário Ramirez, João A Carriço

**Affiliations:** 1 Instituto de Microbiologia and Instituto de Medicina Molecular João Lobo Antunes, Faculdade de Medicina, Universidade de Lisboa, Av. Professor Egaz Moniz, 1649-028 Lisboa, Portugal; 2 National Food Institute, Technical University of Denmark, Kemitorvet, Building 204, DK-2800 Kgs. Lyngby, Denmark

## Abstract

Plasmid ATLAS (pATLAS, http://www.patlas.site) provides an easy-to-use web accessible database with visual analytics tools to explore the relationships of plasmids available in NCBI’s RefSeq database. pATLAS has two main goals: (i) to provide an easy way to search for plasmids deposited in NCBI RefSeq and their associated metadata; (ii) to visualize the relationships of plasmids in a graph, allowing the exploration of plasmid evolution. pATLAS allows searching by plasmid name, bacterial host taxa, antibiotic resistance and virulence genes, plasmid families, and by sequence length and similarity. pATLAS is also able to represent in the plasmid network, plasmid sets identified by external pipelines using mapping, mash screen or assembly from high-throughput sequencing data. By representing the identified hits within the network of relationships between plasmids, allowing the possibility of removing redundant results, and by taking advantage of the browsing capabilities of pATLAS, users can more easily interpret the pipelines’ results. All these analyses can be saved to a JSON file for sharing and future re-evaluation. Furthermore, by offering a REST-API, the pATLAS database and network display are easily accessible by other interfaces or pipelines.

## INTRODUCTION

Plasmids are extrachromosomal genetic elements classified as mobile genetic elements (MGEs) and play a pivotal role in horizontal gene transfer due to their ability to self-replicate and transfer between bacteria (1–3). Plasmids are highly variable in size, mostly circular and, more importantly, they often carry genes providing a selective advantage to the host under specific conditions (1–3). For example, plasmids are key MGEs for the acquisition and spread of antibiotic resistance and may play an important role in the transmission of virulence traits (1,4,5).

Notably, plasmids are considered to be chimeric and modular, as they can easily lose or gain DNA fragments by means of integration or excision of other MGEs, including insertion sequences, transposons, integrons and even other plasmids which may also carry functionally relevant cargo genes (1–3). Furthermore, plasmids can also integrate into the bacterial chromosome which ensures further ways of spread (1–3). This high genetic plasticity clearly indicates that knowledge of plasmids is of utmost importance to understand the dynamics of bacterial communities (1–5). However, the detection of plasmids in high-throughput sequencing (HTS) data is not trivial and it is particularly difficult when using short-read technologies and in metagenomic studies (6).

Currently, many tools are available to assemble or extract plasmids from HTS data, including cBar (7), PLACNET (8), plasmidSpades (9) and Recycler (10), either by searching for specific markers or by assuming that plasmid sequences have distinct characteristics (e.g. plasmid reads tend to have higher sequencing coverage than chromosomal reads, given the higher copy-number of plasmids or contigs resulting from plasmids can be circularized, due to the circular nature of plasmids). However, these assumptions are not always met since plasmids can exist in single-copy in the cell (5) and they can be linear DNA molecules (1–3). Other tools, such as PlasmidFinder (11) and MOB-suite (12), allow plasmid identification using genes defining incompatibility (Inc) groups or replicons (Rep) or relaxase (MOB; (4)) genes, respectively. Another methodology, PlasmidProfiler (Zetner,A., Cabral,J., 52 Mataseje,L., Knox,N., Mabon,P., Mulvey,M. and Van Domselaar,G. (2017) Plasmid Profiler: 53 Comparative Analysis Of Plasmid Content In WGS Data. BioRxiv, https://doi.org/10.1101/121350), uses a combination of read mapping and BLAST+ (13) searches, against the PlasmidFinder database to search for plasmid types, allowing an easier identification of the presence of different plasmids but offering no information regarding their cargo. Regardless of the methodology used to reconstruct or find plasmids in HTS data, the user will struggle to interpret the list of hits and to evaluate the impact of these possible plasmids on the host bacteria.

Currently, there are 13 924 entries in NCBI’s RefSeq plasmid database (14) and there is a lack of tools for browsing this panoply of plasmid sequences. Here, we describe Plasmid Atlas (pATLAS) with the aim of providing an easily accessible visual analytics tool for users to explore the existing plasmids in the NCBI’s RefSeq database and to aid in the identification of plasmids from HTS data. pATLAS enables the visualization and exploration of the metadata associated with all plasmids available in NCBI’s RefSeq database, as well as their putative antibiotic resistance and virulence genes, and plasmid families, through *de novo* annotations based on CARD (15), ResFinder (16), Virulence factors database (VFDB) (17) and PlasmidFinder (11) databases. By offering a REpresentational State Transfer application programming interface (REST API), pATLAS can be easily integrated into pipelines that aim to identify plasmids, in order to take advantage of the database and visualization tools it offers.

## DATABASE CREATION

### Backend

#### Data collection and matrix construction

**Figure 1.**
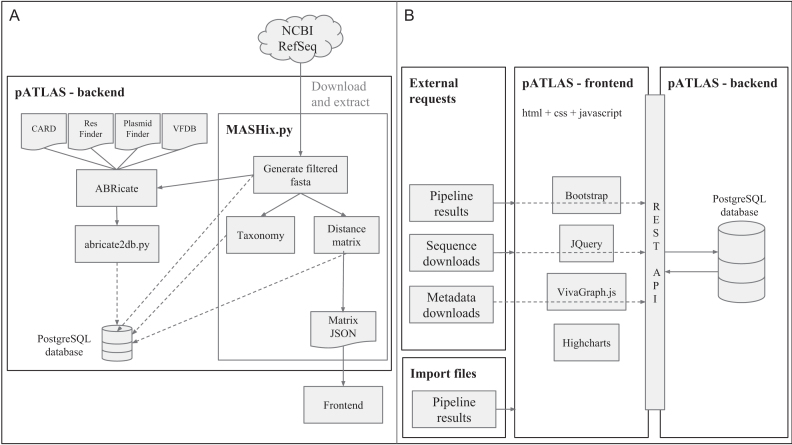
Schematic representation of pATLAS (**A**) database creation in the backend and (**B**) processing of external requests and file imports via the frontend and REST API in the backend.

All sequences available in NCBI’s plasmid RefSeq database (14) (ftp://ftp.ncbi.nlm.nih.gov/refseq/release/plasmid/) were downloaded (last modification: 13 July 2018, 5:27:00 AM) and used to build the pATLAS database and to calculate a network of pairwise relationships. Prior to the analysis, any duplicated sequences or sequences that do not correspond to plasmids, but instead refer to genes found in plasmids, are removed. Therefore, origins of replication and genes corresponding to complete coding sequences were removed by searching the fasta headers for single instances of the keywords ‘origin’ and ‘cds’ (multiple instances of ‘cds’ in the header could potentially be plasmids since these contain multiple genes), and we also retained all sequences that fulfilled these criteria and had the keyword ‘plasmid’ in the fasta header. Furthermore, given the ever growing plasmid RefSeq, manual curation is becoming a daunting task and thus domain experts can submit enquiries for the removal of wrongly included or annotated plasmids from the dataset (crowd curation). All filters used are available in the source code, including crowd curation (https://github.com/B-UMMI/pATLAS). The complete list of the 692 removed sequences is available in Supplementary Table S1 and their length distribution is summarized in Supplementary Figure S1. With this filtered dataset, pairwise distances were calculated using Mash software (v2.0) (18) with the *mash dist* command, a sketch size of 1000 (-s option) and K-mer size of 21 (-k option). Pairwise distances were filtered to exclude distances with a *p-value*>0.05 and a mash distance<0.1. These values were selected in order to retain for display only the pairwise distances that correlate well with an Average Nucleotide Identity (ANI)>0.9 (18) (Supplementary Figures S2 and 3). pATLAS has a REST API that uses a python 3 backend, with flask as a web application framework, which handles the population of the PostgreSQL database with the pairwise distances that meet the above described criteria and saves a JSON file that will be used by the frontend to render the visualization of these relationships (Figure 1).

#### Plasmid metadata

Every plasmid is classified according to the host bacterium in which it is reported in the NCBI RefSeq database, which may not reflect the bacterial species corresponding to the evolutionary origin of the plasmid or reflect its full host range. Species and genera are retrieved from the fasta headers, while families and orders are fetched from NCBI taxonomy (ftp://ftp.ncbi.nlm.nih.gov/pub/taxonomy/) (19). The plasmid size (in base pairs) is calculated from the raw sequence length and, when available, the plasmid name is fetched from the fasta header. Clusters are defined as distinct groups of plasmids connected by links computed from the matrix generated with Mash. Currently pATLAS has 771 clusters and 2915 singletons (plasmids with no links). All these metadata are stored in the PostgreSQL database.

In order to search for antibiotic resistance and virulence genes and also for the targets defining plasmid types in each of the 13 232 plasmids, we used ABRicate v0.8 (https://github.com/tseemann/abricate), with the ResFinder (last update 27 August 2018), CARD (version 2.0.3), VFDB (last update 27 August 2018) and PlasmidFinder (last update 7 September 2018) databases. It is important to highlight that PlasmidFinder databases currently only include plasmids types from *Enterobacteriaceae* and a few Gram-positive bacteria. Then, results were saved in PostgreSQL database, storing the query gene name, accession number, coverage and identity, and the sequence range of the query in the plasmid sequence. In the case of the CARD database, the accession of antibiotic resistance ontology (ARO accession) is also stored in the database. Sequences of all the 13 232 plasmids are stored in pATLAS database so that users are able to download sequences and annotations from the pATLAS website or through requests to the REST API.

#### Local installation

pATLAS can also be locally installed to allow custom built plasmid databases (https://patlas.gitbook.io/docs/api/local_installation). This allows users to explore their own plasmid database in the context of the plasmid RefSeq database available in pATLAS or any subset of it.

To create the default pATLAS database from the current NCBI RefSeq release, the user can take advantage of the scripts available in pATLAS github (https://github.com/B-UMMI/pATLAS) or the nextflow pipeline (https://github.com/tiagofilipe12/pATLAS-db-creation). A docker compose for the latest stable release (currently 1.6.0) of pATLAS (https://github.com/bfrgoncalves/patlas-compose) is also available to easily launch the pATLAS service in a local machine.

### Frontend

pATLAS web page is built in html, css and javascript, using Bootstrap 3 (https://getbootstrap.com/), JQuery version 3.2.1 (https://blog.jquery.com/) and VivaGraphJS (version 0.10.0, https://github.com/anvaka/VivaGraphJS), the latter being a graph visualization library which uses a force-directed layout for rendering the graph. In pATLAS, each node (or vertex) represents a plasmid and each link (or edge) is a relationship between two plasmids from the matrix created with mash and the defined criteria.

Instead of starting pATLAS with the raw JSON file, we allowed the force-directed graph to render until we got an initial visualization of the network and then stored a precomputed session with the positions of all nodes and links. With this approach, instead of computing the network each time the page is loaded, pATLAS displays a precomputed network, speeding up the loading time of the web page as well as displaying a network of plasmids already clearly separating the clusters and showing some of their major features. In the initial page the force layout is paused to prevent an unnecessary drain on the user system resources but can be activated by a simple button click.

Highcharts library (version 6.0.4, https://github.com/highcharts/highcharts) and bootstrap-table version 1.12.1 (http://bootstrap-table.wenzhixin.net.cn/) were used to create interactive plots (such as bar plots, histograms and heatmaps) and tables summarizing metadata for user defined selections, respectively.

### pATLAS usage

#### Web interface

**Figure 2.**
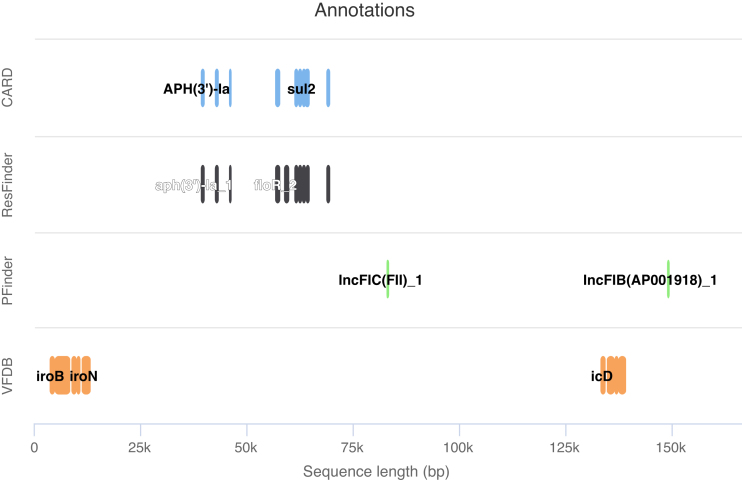
Example of an annotation plot showing the annotations for a given plasmid appearing when its node was clicked in the network of plasmids.

Each pATLAS node can be clicked, thus opening a popover card, that displays the corresponding plasmid metadata: RefSeq accession number (with direct links to NCBI’s website), bacterial host species, sequence length, plasmid name, cluster number and annotations for antibiotic resistance (with direct links to the CARD database - https://card.mcmaster.ca/) and virulence genes, and for plasmid family. When there are annotations, a small plot showing the location of the genes in the plasmid is shown (Figure 2). All these data can be downloaded to a comma-separated values (csv) file for further analysis.

At the bottom center position of the web page there are seven buttons, five of which are enabled by default. These allow users to: interact with the visualization by resuming the force layout (which allows the graph to better separate nodes [plasmids]), select multiple plasmids by clicking in the pen icon and using ‘Shift’ key and mouse drag on a given area, zoom in and zoom out, and center in the node with more links (which is the central node of the default visualization). In the top navigation bar it is possible to search for a given accession number or plasmid name. When that query is found, the card for the plasmid is shown and the visualization is centered on that plasmid.

A series of menus are available through a sidebar, allowing users to take full advantage of the information stored in the pATLAS database. Through these menus users can make selections on the nodes by querying for sequence length, taxa (including species, genera, families and orders), plasmid families, antibiotic resistance and virulence genes. Selections are also possible on links, allowing the user to color them according to mash distance, size ratio (the difference in size between linked plasmids) and percentage of shared sequence (shared hashes). There is also a feature that allows making advanced selections on nodes, by making intersections or unions of different types of queries (e.g. finding selected antibiotic resistances for a given set of taxa).

**Figure 3.**
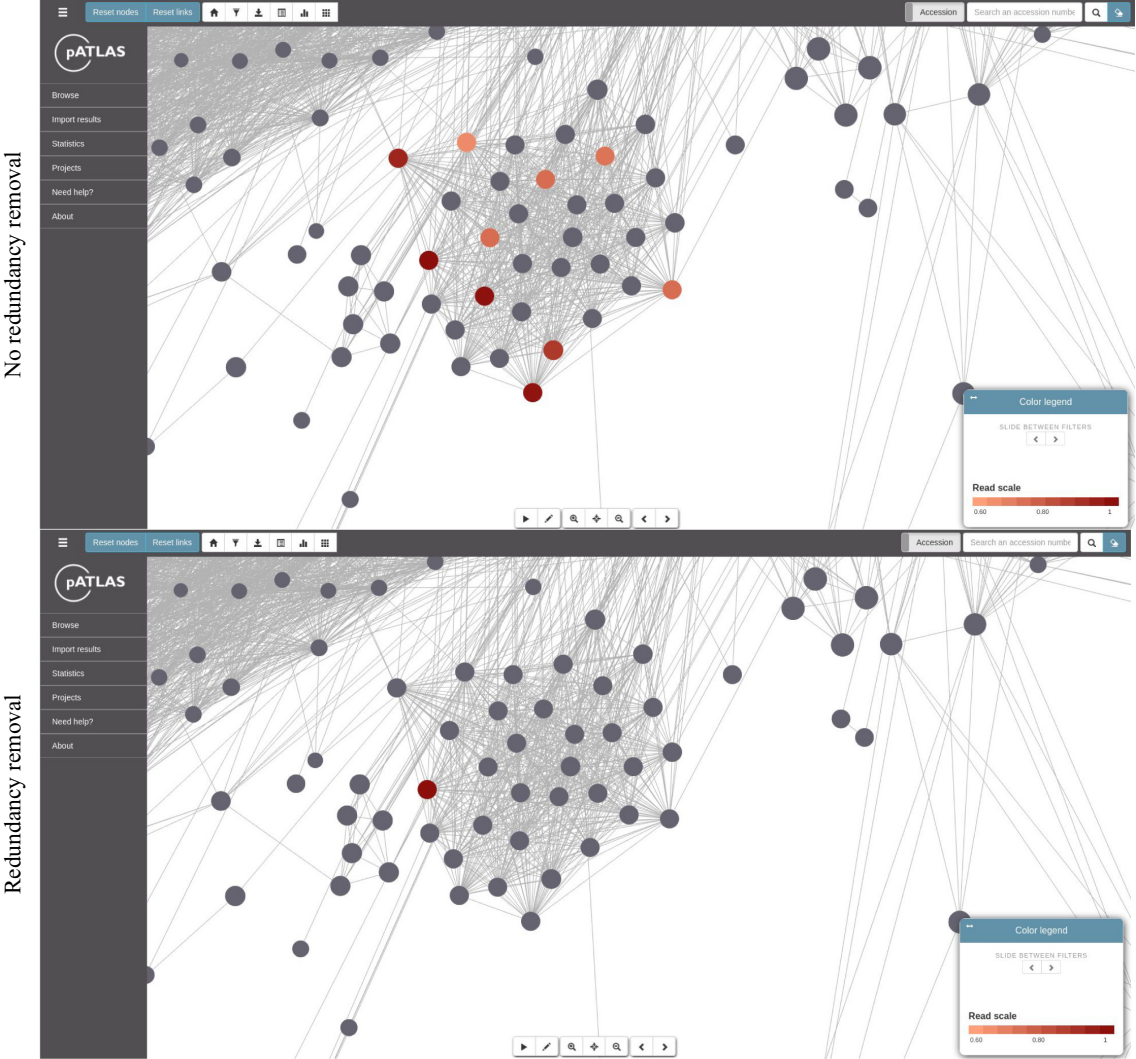
Identifying plasmids related to imported sequence results. Plasmids highlighted in darker red are those with higher percentages of either sequence coverage of (using mapping approaches) or sequence identity with (using mash approaches) imported sequences. If the ‘redundancy removal’ option is used plasmids to remove the highlight on plasmids that are deemed to be less likely to be present in the imported HTS data (with a mash distance of <0.1 between them, using the equations described in this manuscript), compared with other linked plasmids.

It is also possible to import results from external pipelines producing a suitable JSON file (https://patlas.gitbook.io/docs/api/import_api). To assist in the identification of plasmids in HTS data, we have built pipelines that use mapping and mash screen (both using fastq reads), and mash dist (using assemblies in fasta format) based approaches (https://github.com/B-UMMI/pATLASflow), providing, as output, files that can be loaded onto pATLAS. While mapping and mash screen approaches allow users to search plasmids (available in the database) without relying on assembly, which can result in chimeric sequences, mash dist compares assemblies against the sequences available in pATLAS. These are designed so that users run the pipelines locally and then load the resulting JSON file onto the pATLAS website. pATLAS will show the results on the plasmid network, by highlighting the plasmids identified with a red color scheme. Darker red plasmids tend to be the most likely plasmids present, with higher percentages of either sequence coverage (using mapping approaches) or sequence identity with (using mash approaches) imported sequences. (Figure 3). Also, when importing files, users will be asked if they want to remove redundant results. If enabled, this option will remove the highlight on plasmids that are deemed to be less likely to be present in the imported HTS data (with a mash distance of <0.1 between them, using the equations described below), compared with other linked plasmids (Figure 3). For all types of imported results (mapping, mash screen and mash dist from contigs), pATLAS will make pairwise comparisons for all relevant plasmid hits that are linked in the pATLAS network, and a score (*S*) is calculated for each pairwise comparison. In each pairwise comparison, considering plasmids P1 and P2, if the resulting *S* value is positive, P1 is considered to be the most likely plasmid to be present in the HTS data and therefore only P1 is retained for display. Conversely, if the resulting *S* value is negative, P2 is considered the most likely plasmid and only this plasmid is retained for display. If the result is equal to 0, both plasmids (P1 and P2) are considered equally likely to be contained in the HTS data and both are displayed. The calculation of this score is dependent on the approach used.

For the mapping based approach, *S* is calculated using equation A:

\begin{equation*}S\ = P{1_{{\rm cov}}}\ .P{1_{{\rm len}}} - P{2_{{\rm cov}}}.P{2_{{\rm len}}}\left( A \right)\end{equation*}

P1_cov_ and P2_cov_ represent the proportion of each plasmid covered by the HTS reads. P1_len_ and P2_len_ represent each plasmid length in base pairs.

For mash screen approaches, the rational is similar but instead of using the percentage of covered sequence, it uses the percentage identity (ids) retrieved from the *mash screen* output. In this case *S* is calculated as follows:

\begin{equation*}S\ = P{1_{{\rm ids}}}\ .P{1_{{\rm len}}} - P{2_{{\rm ids}}}.P{2_{{\rm len}}}\left( B \right)\end{equation*}

For *mash dist* from contigs, the percentage of shared hashes (sh) needs to be weighted. This is to account for the fact that sequences that share highly similar small subsequences will return high similarity values. These similarity values (idd) are calculated as 1—Mash distances. In this case *S* is calculated as follows:

\begin{equation*}S\ = P{1_{{\rm idd}}}\ .P{1_{{\rm sh}}}.P{1_{{\rm len}}} - P{2_{{\rm idd}}}.P{2_{{\rm sh}}}.P{2_{{\rm len}}}\left( C \right)\end{equation*}

pATLAS browsing capabilities can be used to further filter the results according to user-defined criteria. Users can import multiple result files at once. Upon import, the remaining two buttons on the bottom center of the web page become active to allow the user to cycle between multiple imported files.

Although pATLAS only displays one file at a time onto the network of plasmid relationships, users can use the heat map button available in the top navigation bar to construct a heat map to compare all the files imported, thus allowing comparisons between multiple samples.

**Figure 4.**
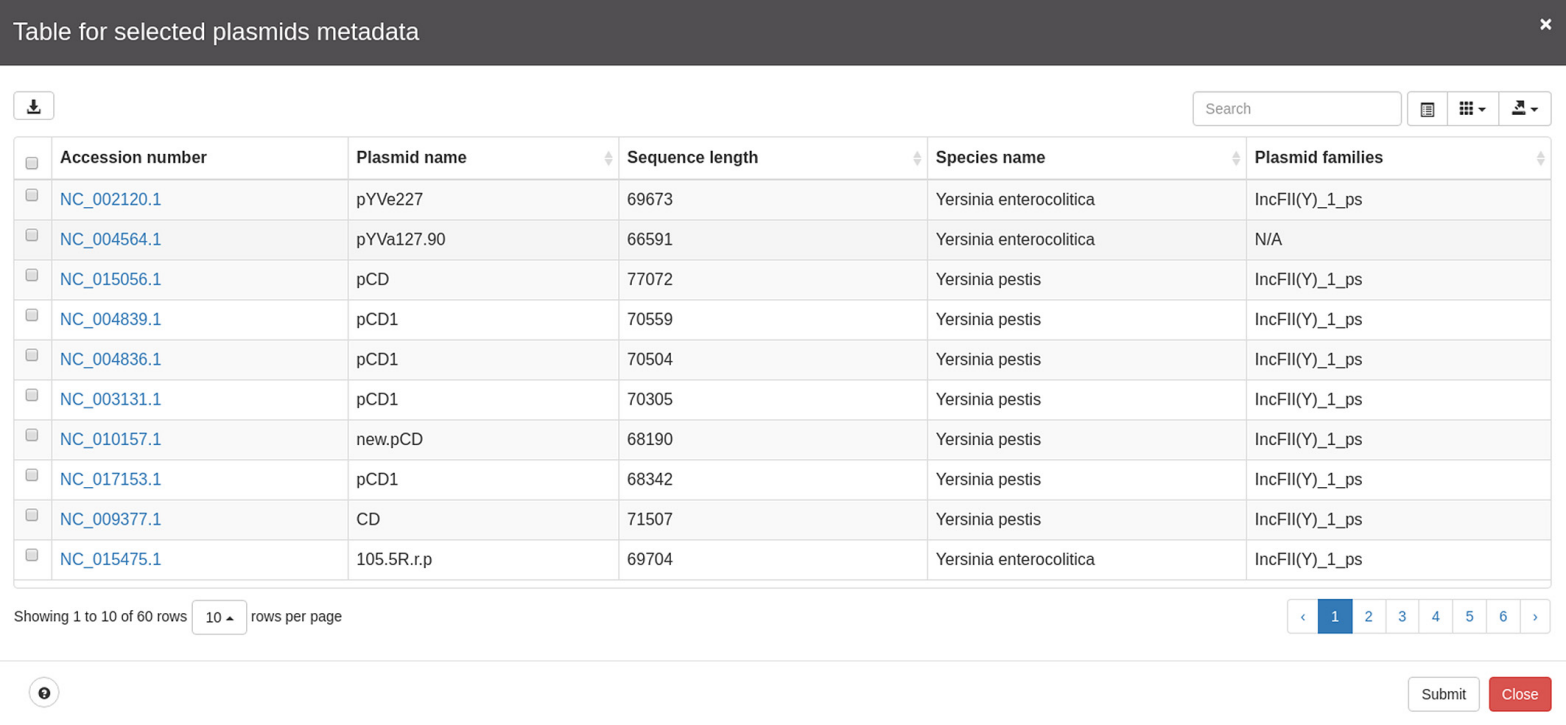
Example of table with metadata available for the selected plasmids in the network of plasmids.

**Figure 5.**
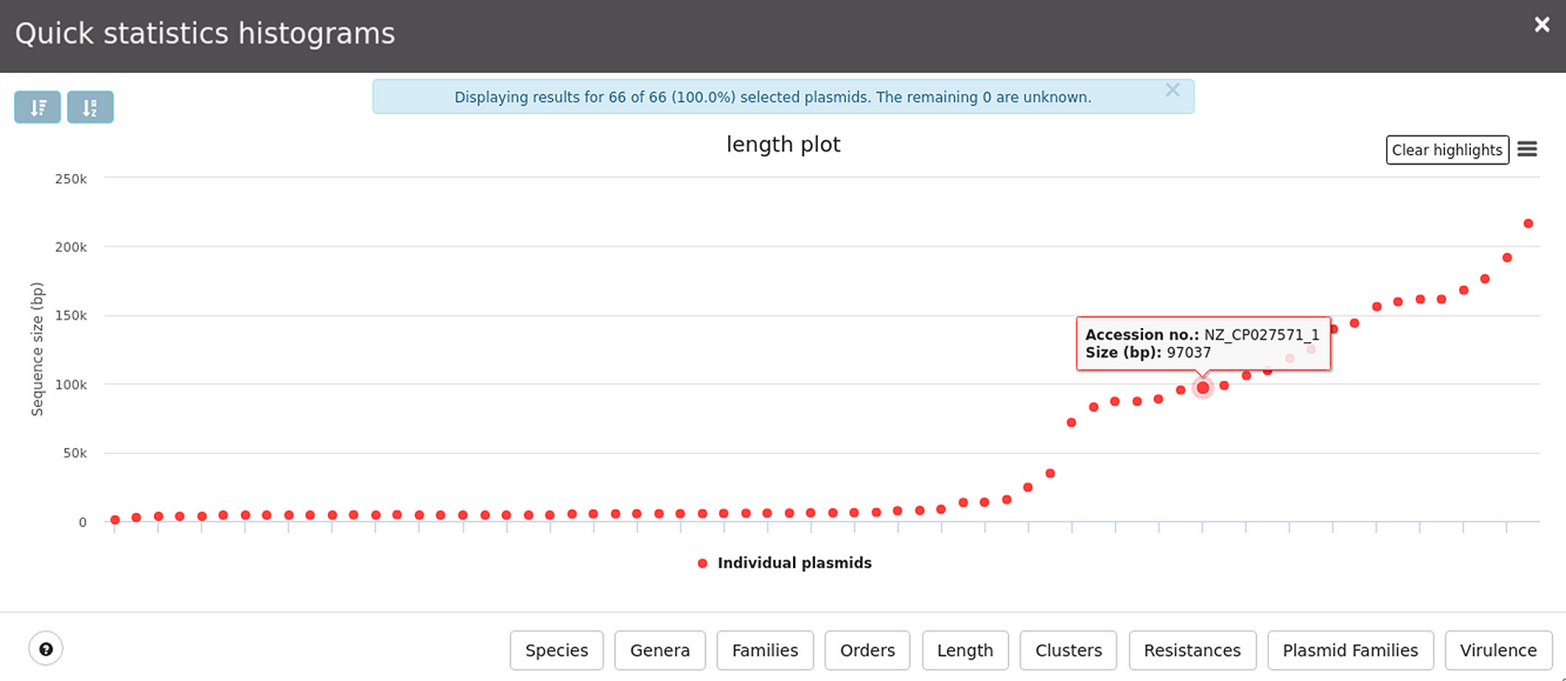
Example bar plot showing length distribution of selected plasmids. The plot is interactive and changes dynamically by highlighting the selection in the various series.

All selections made through these menus are suitable to construct a table displaying plasmid metadata (Figure 4) or to construct specific plots (for instance, users may want to know the size distribution of plasmids present in their selection; for an example see Figure 5). These tables and plots can be exported in commonly used formats such as csv, spreadsheet, and txt, or PDF and png, respectively. Users may also download sequences of interest either by using selections on the plasmid network or using the checkboxes available in the table for further refinement of the plasmids of interest.

The website can also create projects, which allows to save any selection made in pATLAS for later inspection or even for sharing with other researchers. Projects are saved as a JSON file which is downloaded to the user’s computer.

### Application programming interface

The user can interact with pATLAS both through the web interface and programmatically, by taking advantage of the REST API, to query the PostgreSQL database in order to obtain data.

pATLAS allows users to download metadata as well as sequence data associated with a given plasmid without using the pATLAS frontend (examples on how to request these downloads can be found here: https://patlas.gitbook.io/docs/api/download_api). These requests will output a parsable object/JSON with all metadata (including plasmid sequence data, antibiotic resistance and virulence genes, and plasmid families) or fasta file sequences associated with the queried accession numbers, respectively.

The REST API can also receive requests with results from pipelines that follow a similar structure to the JSON files imported through the pATLAS menus (examples of these requests can be found here: https://patlas.gitbook.io/docs/api/requests_api). Following a request it will return a unique uniform resource locator (URL) in which the results can be visualized, similarly to the import from file function. These URLs contain a hash generated from the queried accession number list, and their results will be stored in the pATLAS database for 24 h, after which they will be deleted from the database. This will not store any user specific data (such as logins, raw sequencing data, project names) but will store simple keys of accession numbers and values including percentages (e.g. percentage of identity), integer values (e.g. estimated copy number) and contig identification strings, that can, for example, be the original names given by the assemblers or aliases defined by the pipelines that generate these JSON files.

The objective of these requests is to allow other tools to use the pATLAS database (to download its sequences, sequence metadata and annotations) or to visualize results in the pATLAS network using a simple temporary link as a response to a request.

## CONCLUSION

pATLAS allows the user to explore the plasmid NCBI RefSeq database through queries by name, accession number, plasmid size, taxa of bacterial host, plasmid family and antibiotic resistance and virulence genes. The latter are independently annotated when creating the database, using standard tools, thus ensuring consistency across all plasmids. This can, for example, allow users to quickly identify all plasmids carrying a given antibiotic resistance gene found in a given species or genus, and quickly obtain summarized metadata, download the selected plasmids and generate plots, something that is not easily achieved without pATLAS. Moreover, the plasmids of interest will be represented within a context of related plasmids, which allows the user to easily identify variants found in other species or lacking a specific antibiotic resistance gene. pATLAS also provides a platform to facilitate the identification of plasmids contained within HTS data.

State-of-the-art tools focus on detecting plasmid types (such as Inc groups) or which plasmids may be contained in HTS data (5,20). Also, plasmid typing methodologies do not identify important traits, particularly in the context of bacterial pathogens, such as antibiotic resistance genes. On the other hand, assembly based and mapping-based approaches, coupled with databases of known plasmids can identify plasmids; however they ignore the high modularity of plasmids and the possible close relationship between hits. Navigating and exploring these lists of hits is often tedious, frustrating and time consuming. To overcome this, pATLAS offers a visual analytics tool to explore the existing plasmid database and to assist in exploring the results of plasmid-finding approaches from HTS data. By implementing a quick method to select the most relevant results (i.e. by using the option to remove redundant results based on the algorithms described) and by creating a rich browsing environment that can be used to further sift through and explore the results, pATLAS will contribute to the accuracy of HTS approaches for plasmid identification in both single strain and metagenomics HTS data.

## DATA AVAILABILITY

pATLAS is freely available at www.patlas.site. Its source code, including the web interface, REST API and scripts necessary to create the database are hosted at https://github.com/B-UMMI/pATLAS and the documentation can be found in https://patlas.gitbook.io/docs.

## Supplementary Material

Supplementary Data
