# Supplementary material for: Plasmid ATLAS: plasmid visual analytics and identification in high-throughput sequencing data
Source: Nucleic Acids Res. 2018 Nov 5;47(Database issue):D188–94. doi: 10.1093/nar/gky1073 (PMC6323984; doi:10.1093/nar/gky1073)
Supplement: Supplementary Data [file gky1073_supplemental_files.zip › supplementary_material_legends.docx]

Table S1 - List of all removed entries per category and the reason for removal.

Figure S1 - Distribution of sequence length of all the removed sequences per reason for removal (presence of the keyword “cds” and “origin”, and “crowd curation”(comprising 1 gene, 1 chromosome and 2 synthetic plasmids). In the current version of the plasmid NCBI RefSeq database (corresponding to pATLAS v1.6.0) there are no duplicated entries and thus there is no box plot for that category.

Figure S2 - Comparison of pATLAS links (1 - mash dist, including only values >0.9) with fastANI (<https://github.com/ParBLiSS/FastANI>) for all links between plasmids with more than 10,000 bp (comprising 297,783 links). The values of mash and fastANI range from 0, no similarity, to 1, 100% identity. The figure represents the similarity value between pairs of plasmids (represented by links in pATLAS) . The vast majority of pATLAS links (pairwise comparisons) have ANI > 0.9. Only a small number links considered in pATLAS (1 - mash dist > 0.9) have ANI values <0.9, confirming that mash is adequate for the purposes of pATLAS.

Figure S3 - Comparison of Mash (1 - mash dist) with ANIb (<https://github.com/widdowquinn/pyani>) for 1,000 randomly selected links. The values of mash and ANIb range from 0, no similarity, to 1, 100% identity. The vast majority of pATLAS links (pairwise comparisons) have ANIb > 0.9. Only a small number links considered in pATLAS (1 - mash dist > 0.9) have ANIb values inferior to 0.9, confirming that mash is adequate for the purposes of pATLAS.
